# Enhancing the Total Phenolic Content and Antioxidants of Lemon Pomace Aqueous Extracts by Applying UV-C Irradiation to the Dried Powder

**DOI:** 10.3390/foods5030055

**Published:** 2016-08-23

**Authors:** Konstantinos Papoutsis, Quan V. Vuong, Penta Pristijono, John B. Golding, Michael C. Bowyer, Christopher J. Scarlett, Costas E. Stathopoulos

**Affiliations:** 1 School of Environmental and Life Sciences, The University of Newcastle, P.O. Box 127, Ourimbah 2258, NSW, Australia; Konstantinos.Papoutsis@uon.edu.au (K.P.); vanquan.vuong@newcastle.edu.au (Q.V.V.); penta.pristijono@newcastle.edu.au (P.P.); john.golding@dpi.nsw.gov.au (J.B.G.); michael.bowyer@newcastle.edu.au (M.C.B.); c.scarlett@newcastle.edu.au (C.J.S.); 2 NSW Department of Primary Industries, Locked Bag 26, Gosford 2250, NSW, Australia; 3 Division of Food and Drink, School of Science, Engineering and Technology, University of Abertay, Dundee DD1 1HG, UK

**Keywords:** UV treatment, proanthocyanidins, antioxidants, lemon waste, dried powder

## Abstract

Several studies have shown that UV-C (ultraviolet C) irradiation promotes the bioactive compounds and antioxidants of fresh fruits and vegetables. The aim of this study was to apply UV irradiation in dried lemon pomace powder for enhancing its phenolic content and antioxidant properties, thus more bioactive compounds should be available for extraction and utilization. Lemon pomace dried powder was placed under a UV lamp and treated with dosages of 4, 19, 80 and 185 kJ·m^−2^, while untreated powder was used as a control. UV-C irradiation significantly affected the total phenolic content, total flavonoid content, proanthocyanidins, and antioxidant capacity measured by cupric reducing antioxidant capacity (CUPRAC) and ferric reducing antioxidant power (FRAP) of the lemon pomace dried powder, while it did not affect the vitamin C content. UV-C irradiation of 19 kJ·m^−2^ resulted in 19% higher total phenolic content than the control, while UV-C irradiation of 180 kJ·m^−2^ resulted in 28% higher total flavonoid content than the control. The antioxidant capacity was reduced when UV-C irradiation more than 4 kJ·m^−2^ was applied. The results of this study indicate that UV-C treatment has the potential to increase the extraction of bioactive compounds of dried lemon pomace at relatively high dosages.

## 1. Introduction

Lemon (*Citrus limon* L.) is an important Citrus species with a strong commercial value and generates a large amount of waste. The main component of this waste is the peel, which accounts for 50% to 65% of the whole fruit weight [1]. Lemon peel contains phenolic compounds, such as flavonoids (flavanones, flavonols, flavones), phenolic acids (ferulic, p-coumaric and sinapic acids), as well as vitamin C (ascorbic acid) [1,2], which have been linked to antimicrobial [3], anticancer [4], and antioxidant properties [5,6].

Phenolic compounds are the most abundant secondary metabolites synthesized by plants through the shikimate pathway [7] as a response to external stresses, such as ultraviolet radiation, wounding, aggression by pathogens, parasites and predators, in addition, they contribute to the color of plants [8,9]. Several treatments, such as heat treatment using electric oven [10,11], electron-beam irradiation [12], and microwave treatment, have been applied to Citrus peel powder for the enhancement of its phenolic content and antioxidant capacity [13].

UV-C (ultraviolet C) is an electromagnetic, non-ionizing radiation, which has been applied to fruits and vegetables in order to prolong their storage life by inactivating some pathogens [14,15] or retarding their senescence [16]. Perkins-Veazie et al. [17] reported that UV-C radiation of 2 kJ·m^−2^ reduced ripe rot (*Colletotrichum acutatum*, syn. C. gloeosporioides) in blueberries. Arcas et al. [18] mentioned that UV radiation reduced, by up to 45%, the growth of *Penicillium digitatum* in *Citrus aurantium*, which was attributed to the accumulation of flavonoids in treated fruits.

Apart from extending the storage life of fruits and vegetables, UV-C irradiation may promote their bioactive compounds and antioxidants [19,20,21]. Alothman et al. [20] reported that the total phenolic content of banana and guava increased significantly after 10 min UV-C treatment. Perkins-Veazie et al. [17] reported that the total anthocyanin content and antioxidant capacity measured by Ferric reducing antioxidant power (FRAP) of one blueberry variety (*Vaccinium corymbosum*) increased with the UV-C treatment dosage, whereas the total phenolic content was not significantly affected by the UV-C treatment. Liu, et al. [22] found that UV-C irradiation of 4 or 8 kJ·m^−2^ promoted the accumulation of total flavonoids and increased the antioxidant activity of tomato fruit. In addition, Bravo et al. [23] showed that UV treatment significantly increased the total phenolic content and the antioxidant capacity of tomatoes during eight-day storage, compared to untreated samples, but it did not have a clear effect on individual phenolic compounds.

As has been already reported, the postharvest application of UV-C irradiation promotes the antioxidant capacity and bioactive compounds of fruits and vegetables. Since enzymes are still active in freeze dried materials [24,25], enhancing the phenolic and antioxidant capacity of Citrus powder, more bioactive compounds might be available for the extraction, isolation, and utilization. To the best of our knowledge, there have been no studies examining the effect of UV-C irradiation on dried Citrus pomace powder regarding the phenolic compounds and antioxidants. Therefore, the aim of this study was to examine the effects of UV-C irradiation on bioactive compounds and antioxidants of dried lemon pomace powder aqueous extracts.

## 2. Materials and Methods

### 2.1. Plant Material

Lemon (*Citrus limon* L.) waste, including peel and seeds, was provided by a commercial juicing factory in Kulnura (NSW Australia), in September, 2015. After collection, the seeds were removed and the remaining peels were freeze dried (FD3 freeze dyer, Thomas Australia Pty Ltd., Seven Hills, NSW, Australia). The dried waste was ground using a commercial blender (John Morris Scientific, Chatswood, NSW, Australia) and the material passed through a 1.4-mm sieve (1.4 mm EFL 2000; Endecotts Ltd., London, England) was stored at −18 °C for further use. The water activity of the dried lemon pomace powder was 0.30 ± 0.01 (mean average ± standard deviation).

### 2.2. UV-C Treatment of the Lemon Pomace Dried Powder

**Figure 1:**
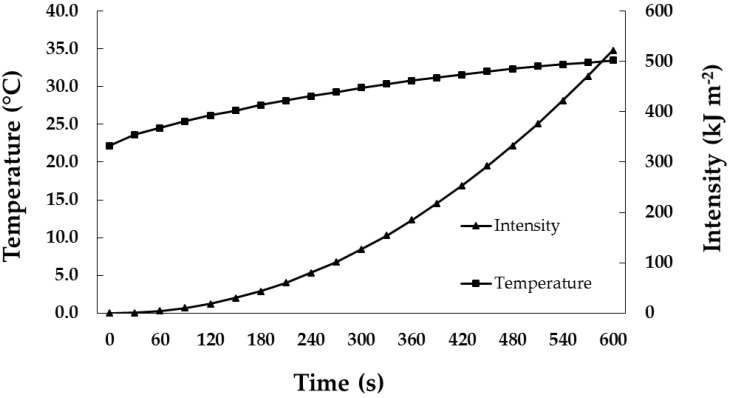
UV-C (ultraviolet C) intensity and temperature overtime.

A custom made light-proof box, fitted with two germicidal lamps (Sahkyo Denki Co., Ltd. G20T10 20 Watt, low pressure mercury, Kanagawa, Japan), was used. A SED008/W detector with a PIR Irradiance Calibration at 254 nm was used to monitor the UV-C dosage. This was connected to an International Light Technologies 1700 series research radiometer that recorded cumulative exposure over time. The sensor was placed inside the box with the samples during treatments. The UV-C light was turned off manually once exposure time had reached the required treatment units. Two grams of dried lemon pomace powder was placed 7.5 cm under the UV lamp and treated for 60, 120, 240, and 360 s, which were equal to the dosages of 4, 19, 80, and 185 kJ·m^−2^, respectively (Figure 1), while untreated powder was used as a control. After UV-C treatments, the control and treated powder were stored for 24 h at −18 °C.

### 2.3. Extraction Process

An ultrasonic bath (Soniclean, 220 V, 50 Hz and 250 W, Soniclean Pty Ltd., Thebarton, Australia) with pre-set conditions at a temperature of 30 °C, time of 20 min, and power of 60 W was used. Water used as a solvent at sample-to-solvent ratio of 1:100 g·mL^−^^1^. After completion of the extraction, the extracts were filtered through Whatman no. 1 filter paper under ambient temperature and then stored at −18 °C for further phytochemical analysis.

### 2.4. Total Phenolic Content (TPC)

The total phenolic content was measured, as described by Vuong et al. [26]. Briefly, 5 mL of 10% (*v/v*) Folin-Ciocalteu reagent was mixed with 1 mL of sample and 4 mL of 7.5% (*w/v*) Na_2_CO_3_ and incubated under dark at room temperature for 1 h prior to the absorbance being measured at 760 nm by a UV spectrophotometer (Varian Australia Pty Ltd., Victoria, Australia). The results were expressed as mg gallic acid equivalents per g of sample dry weight (mg·GAE·(g·dw)^−^^1^).

### 2.5. Total Flavonoid Content (TF)

The total flavonoid content was measured, as described by Zhishen et al. [27]. Briefly, 2 mL of H_2_O was mixed with 0.15 mL of 5% (*w/v*) NaNO_2_ and 0.5 mL of sample and left at room temperature for 6 min. Then, 0.15 mL of 10% (*w/v*) AlCl_3_ was added and left at room temperature for 6 min. Subsequently, 2 mL of 4% (*w/v*) NaOH and 0.7 mL of H_2_O were added and the mixture was left at room temperature for 15 min before the absorbance was measured at 510 nm. The results were expressed as mg of catechin equivalents per g of sample dry weight (mg·CE·(g·dw)^−^^1^).

### 2.6. Proanthocyanidins

The proanthocyanidin content was measured according to the method described by Li et al. [28]. Three milliliters of Vanillin (4% *w/v*) were mixed with 0.5 mL of sample, followed by 1.5 mL of concentrated HCl. The mixture was left for 15 min at room temperature before the absorbance was measured at 500 nm. The results were expressed as mg of catechin equivalents per g of sample dry weight (mg·CE·(g·dw)^−^^1^).

### 2.7. Vitamin C (Ascorbic Acid)

Total vitamin C was determined as described by Vuong et al. [29], with some modifications. A reagent solution was prepared by mixing 500 mL of 0.6 M sulfuric acid with 5.3218 g of Sodium Phosphate and 2.471 g of ammonium molybdate. The reagent (3 mL) was mixed with 0.3 mL of sample and incubated at 95 °C for 90 min in a water bath. After incubation, they were cooled in water for 5 min before the absorbance was measured at 695 nm. The results were expressed as mg ascorbic acid equivalents per g (mg·AAE·g^−^^1^).

### 2.8. Antioxidant Capacity

#### 2.8.1. 2,2-Diphenyl-1-picrylhydrazyl (DPPH) Radical Scavenging Capacity

A DPPH assay was performed as described by Thaipong et al. [30], with some modifications. A stock solution was prepared and stored at −20 °C until use. The working solution was then freshly prepared by mixing 10 mL of stock solution with 45 mL of methanol to obtain an absorbance of 1.1 ± 0.02 (λ = 515 nm). A volume of 2.85 mL of working solution was mixed with 0.15 mL of sample and left under darkness at room temperature for 30 min before measuring the absorbance at 515 nm. The results were expressed as mg of trolox equivalents per g of dry weight (mg·TE·(g·dw)^−^^1^).

#### 2.8.2. Ferric Reducing Antioxidant Power (FRAP) Assay

FRAP was measured as described by Thaipong et al. [26], with some modifications. A working FRAP solution was prepared by mixing 300 mM acetate buffer, 10 mM TPTZ (2,4,6-tripyridyl-s-triazine) in 40 mM HCl and 20 mM FeCl_3_ at a ratio of 10:1:1. Working FRAP solution (2.85 mL) was mixed with 0.15 mL of sample and incubated at room temperature under dark for 30 min before its absorbance was measured at 593 nm. The results were expressed as mg trolox equivalents per g of dry weight (mg·TE·(g·dw)^−^^1^).

#### 2.8.3. Cupric Reducing Antioxidant Capacity (CUPRAC) Assay

CUPRAC was determined as described by Apak et al. [31], with some modifications. CuCl_2_ (1 mL) was mixed with 1 mL of neocuproine, 1 mL of NH_4_Ac and 1.1 mL of sample. The mixture was incubated at room temperature for 1.5 h before measuring the absorbance at 450 nm. The results were expressed as mg of trolox equivalents per g of sample dry weight (mg·TE·(g·dw)^−^^1^).

### 2.9. Statistical Analysis

The one-way ANOVA (Completely Randomized Design (CRD)) was conducted by using SPSS software (version 23, IBM Crop., Armonk, NY, USA). The means were compared by the Least Significance Difference (LSD) at *p <* 0.05. Data were reported as means ± standard deviations. Each experiment was performed in triplicate.

## 3. Results and Discussion

### 3.1. Total Phenolic Content (TPC)

**Figure 2:**
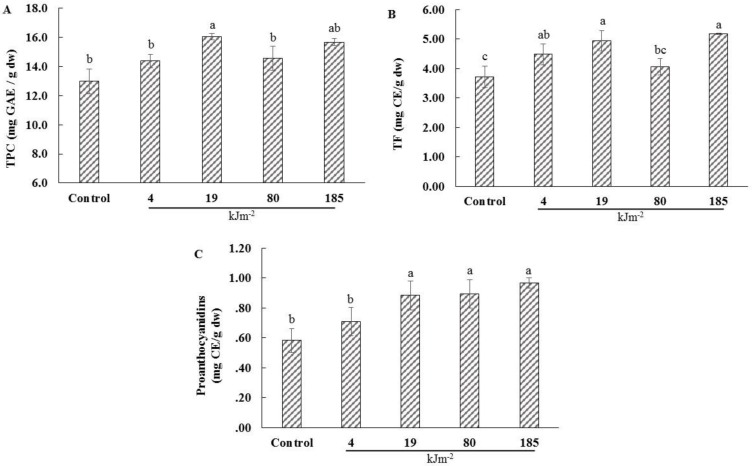
The effect of different UV-C irradiations on the total phenolic content (TPC) (**A**), total flavonoid content (TF) (**B**), and proanthocyanidins (**C**), of the aqueous lemon pomace extracts. The values are the mean of three replications. Data not sharing the same superscript letter are significantly different at *p* < 0.05.

UV-C treatment had a significant effect on the total phenolic content of the dried lemon pomace powder aqueous extracts (Figure 2A) (*p* < 0.05, CV = 5.46%). Lemon pomace powder exposed to 19 kJ·m^−2^ had the highest total phenolic content (16.05 mg·GAE·(g·dw)^−^^1^), while the control had the lowest (12.97 mg·GAE·(g·dw)^−^^1^). These results are in agreement with Kim et al. [12], who mentioned that electron-beam irradiation increased the total phenolic content of dried Citrus pomace powder. González-Aguilar et al. [32] showed that UV-C irradiation had a significant effect on the total phenolic compounds of freshly cut mangoes. Mangoes treated for 10 min had the highest total phenolic content compared to the 5-, 3-, 1-min treatments and the control. The increased total phenolic content of the treated powder could be attributed to the increased phenylalanine ammonia lyase activity, which is a key enzyme for phenolic compound synthesis in plant tissues, since it catalyzes the conversion of l-phenylalanine to ammonia and trans-cinnamic acid, which is the initial step for the synthesis of polyphenols [20]. Stevens et al. [33] reported that UV-C irradiation increased the activity of phenylalanine ammonia lyase in peaches. UV-C dosage higher than 19 kJ·m^−2^ resulted in a reduction of TPC. This could be attributed to the reduction of some phenolic compounds, such as phenolic acids or flavonoids. These results are in accord to Bravo et al. [23], who showed that UV-C irradiation of tomatoes enhanced their content in some phenolic acids and flavonoids; however, the content of some phenolic acids and flavonoids declined when the dosage increased. Indeed, the studies mentioned above have been conducted in fresh materials; however, according to previous studies, enzymes are still active in dried materials. Overall, among the different UV-C intensities examined, the dosage of 19 kJ·m^−2^ resulted in 19% higher total phenolic content of the dried lemon pomace powder aqueous extract, compared to the control.

### 3.2. Total Flavonoid Content (TF)

UV-C treatment had a significant effect on the total flavonoid content of the dried lemon pomace powder aqueous extracts (Figure 2B) (*p* < 0.05, CV = 9.28%). As the UV-C dosage increased, the total flavonoid content increased. Lemon pomace powder exposed to 185 and 19 kJ·m^−2^ had the highest total flavonoid content (5.19 and 4.94 mg·CE·(g·dw)^−^^1^), while the control had the lowest (3.72 mg·CE·(g·dw)^−^^1^). These results are in accord with Arcas et al. [18], who supported that the levels of two flavonoid compounds (naringin and tangeretin) of *Citrus aurantium* fruit increased during storage, after exposure at UV-C irradiation for 2 h. González-Aguilar et al. [32] have also reported that UV-C irradiation affected the flavonoid content of freshly cut mangos. Flavonoids are synthesized by the plants as a response to different stresses, such as UV irradiation, water stress, or temperature [34]. The accumulation of the flavonoid compounds in the dried lemon pomace powder could be attributed to the activation of enzymes, which are responsible for the synthesis of compounds, such as phenylalanine ammonia lyase (PAL) and chalcone synthase (CSH), by UV-C irradiation [35]. Previous studies have shown that enzymes are still active in dried plant tissues. Lam et al. [25] have reported that the retention of PAL activity in freeze-dried wheat seedling tissues, following three months of storage at −20 °C, ranged from 62% in the leaf to 89% in root/residual seed tissue. Lester et al. [24] showed that enzyme activities of freeze-dried fruit tissues were significantly lower or higher than those in fresh fruits.

### 3.3. Proanthocyanidins

Proanthocyanidins are polyphenols derived from the flavonoid pathway. They are oligomeric flavonoids consisting of a few monomeric units, such as catechins, epicatechins, and gallocatechins, and have been linked to antioxidant, antibacterial, antiviral, and anticancer activities [36,37].

UV-C treatment had a significant effect on the proanthocyanidin content of the dried lemon pomace powder aqueous extracts (Figure 2C) (*p* < 0.05, CV = 10.28%). Dried lemon pomace powder exposed to 185, 80, and 19 kJ·m^−2^ had higher proanthocyanidin content (0.97, 0.89, and 0.88 mg·CE·(g·dw)^−^^1^, respectively) compared to the control (0.58 mg·CE·(g·dw)^−^^1^). These results are in agreement with Cetin [38], who mentioned that UV-C irradiation stimulated the proanthocyanidin synthesis of *Vitis vinifera* L. Öküzgözü callus cultures. Indeed, proanthocyanidins consist of monomeric units. The accumulation of the proanthocyanidins in dried lemon pomace powder could be attributed to the synthesis of flavonoid compounds, such as catechins [38], which are present in Citrus peels [13], as a response to the UV-C light. Overall, UV-C treatment could be used for the enhancement of the proanthocyanidin content of dried lemon pomace powder.

### 3.4. Vitamin C (Ascorbic Acid)

**Figure 3:**
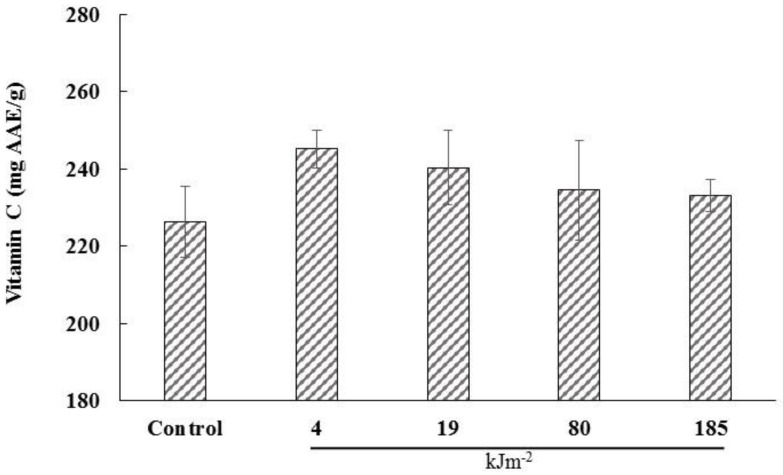
The effect of different UV-C irradiations on the vitamin C (ascorbic acid) content of the aqueous lemon pomace extracts. The values are the mean of three replications.

UV-C treatment had no effect on the vitamin C content of the dried lemon pomace powder aqueous extracts (Figure 3) (*p* < 0.05, CV = 5.16%). Cantos et al. [39] and George et al. [40] reported that UV-C treatment had no effect on the vitamin C content of grape berries and freshly cut mangos, respectively. On the other hand, Lemoine et al. [41] supported that UV-C treated broccoli florets had higher vitamin C content than the control. To sum up, UV-C irradiation had no adverse effect on the vitamin C of dried lemon pomace powder. This might indicate that UV-C irradiation is not implicated in the vitamin C pathway synthesis.

### 3.5. Antioxidant Capacity

The effect of the UV-C irradiation on the antioxidant capacity of dried lemon pomace powder aqueous extracts was measured using three antioxidant assays: CUPRAC, FRAP, and DPPH, since each antioxidant assay has its own advantages and limitations.

**Figure 4:**
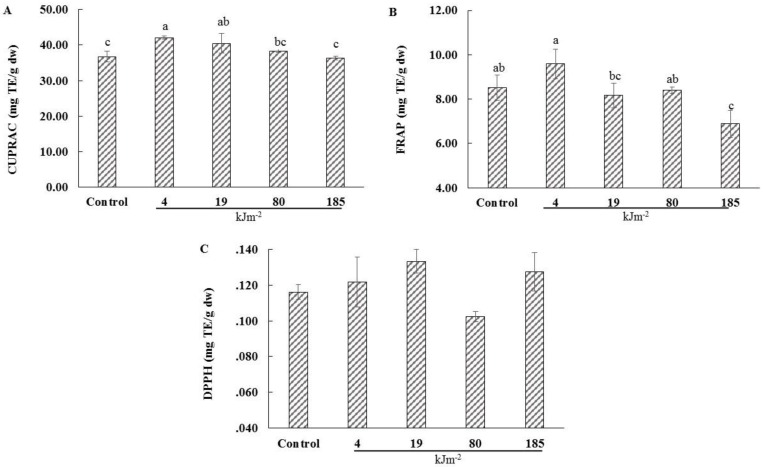
The Effect of different UV-C irradiations on the antioxidant capacity (Cupric reducing antioxidant capacity (CUPRAC) (**A**), Ferric reducing antioxidant power (FRAP) (**B**) and 2,2-diphenyl-1-picrylhydrazyl (DPPH) (**C**)) of the aqueous lemon pomace extracts. The values are the mean of three replications. Data not sharing the same superscript letter are significantly different at *p* < 0.05.

UV-C treatment had a significant effect on antioxidant capacity, as measured by CUPRAC and FRAP assays (*p* < 0.05, CV = 5.05% and 8.97%, respectively) (Figure 4A,B), while no effect on the antioxidant capacity was measured by the DPPH assay (Figure 4C). For CUPRAC, the highest antioxidant capacity was detected when the dried lemon pomace powder was exposed to 4 kJ·m^−2^ (42.13 mg·TE·(g·dw)^−^^1^), which is 13% higher than the control, while exposure at higher UV-C irradiation resulted in a decrease of antioxidants. For FRAP, the highest antioxidant capacity was detected at 4 kJ·m^−2^ (9.56 mg·TE·(g·dw)^−^^1^), while exposure at higher irradiation led to a decrease of antioxidants. Perkins et al. [17] reported that 2 or 4 kJ·m^−2^ UV-C treatment increased the antioxidants (FRAP) of blueberries (*Vaccinium corymbosum*, cvs. Collins, Bluecrop). The increase in the antioxidant capacity could be attributed to the synthesis of some compounds, such as phenols, flavonoids, or other non-phenolic compounds, such as enzymes, since previous studies have found that antioxidant enzymes are still active in dried plant tissues [24]. The accumulation of antioxidant enzymes in strawberry fruit after UV-C treatment has been reported [19]. Indeed, UV irradiation results in the formation of reactive oxygen species, since it is an abiotic stress for plant tissues [42]. The reduction in antioxidant capacity occurring at higher UV-C irradiations could be due to the reaction of the antioxidant compounds with the reactive oxygen species produced by the UV stress [42]. Overall, appropriate UV-C treatment may enhance the antioxidant capacity of the dried lemon pomace powder; however, the applied dosage should be carefully chosen.

## 4. Conclusions

UV-C irradiation had a significant effect on the total phenolic content, total flavonoid content, proanthocyanidins, and antioxidant capacity, measured by CUPRAC and FRAP, of dried lemon pomace powder, while it did not affect the vitamin C content and antioxidant capacity measured by DPPH. UV-C irradiation of the dried lemon pomace powder with 19 kJ·m^−2^ resulted in aqueous extracts with 19% more total phenolic content than the control, while UV-C irradiation of 180 kJ·m^−2^ resulted in 28% and 40% higher total flavonoid and proanthocyanidin content, respectively, compared to the control. The antioxidant capacity reduced when UV-C irradiation greater than 4 kJ·m^−2^ was applied. The results of this study indicate that UV-C treatment should be effectively applied in the dried lemon pomace powder for enhancing its total phenolic content and antioxidant capacity. Studies examining the effect of UV-C irradiation on the total and individual phenolic compounds and antioxidants of several fruit waste dried powders should be conducted.
